# Neural Repair and Neuroprotection with Stem Cells in Ischemic Stroke

**DOI:** 10.3390/brainsci3020599

**Published:** 2013-04-23

**Authors:** Laura L. Stone, Andy Grande, Walter C. Low

**Affiliations:** 1 Graduate Program in Neuroscience, University of Minnesota, Minneapolis, MN 55455, USA; E-Mail: stone337@umn.edu; 2 Department of Neurosurgery, University of Minnesota, Minneapolis, MN 55455, USA; E-Mail: grande@umn.edu; 3 Stem Cell Institute, University of Minnesota, Minneapolis, MN 55455, USA

**Keywords:** ischemia, ischemic brain injury, neuroprotection, stem cells, stroke

## Abstract

Stem cells have been touted as a potential source of cells for repair in regenerative medicine. When transplanted into the central nervous system, stem cells have been shown to differentiate into neurons and glia. Recent studies, however, have also revealed neuroprotective properties of stem cells. These studies suggest that various types of stem cells are able to protect against the loss of neurons in conditions of ischemic brain injury. In this article, we discuss the use of stem cells for ischemic stroke and the parameters under which neuroprotection can occur in the translation of stem cell therapy to the clinical setting.

## 1. Introduction

Stroke is one of the most common causes of death in the United States, with over 750,000 cases per year in the US [1]. Moreover, those who suffer stroke and survive do so at great cost to society, with direct costs estimated at $18.8 billion per year [1]. Despite the high prevalence of stroke, there remain only limited options for therapy in the clinic, none of which are effective for restoring lost neurological function. In addition, available treatments are only effective at the acute phase of stroke. The lack of effective treatments for such a pervasive disease have made novel approaches to stroke therapy a focus of both preclinical and clinical research in recent years. One of the most prominent approaches to stroke therapy that is emerging from this body of research is the use of exogenous stem cells as a therapy for ameliorating stroke deficits and restoring neurological function.

## 2. Treatment

Currently available treatments for ischemic stroke focus on revascularization—removal of the clot in the brain to restore normal blood flow, while restoring normal cranial pressure to reduce damage due to swelling. The only currently proven therapy for stroke is tissue plasminogen activator (tPA) which disrupts the clot, but is only effective if administered via IV within 4.5 h of the stroke occurrence, or delivered via IA administration up to 6 h following stroke [2]. 

In addition, there are surgical methods of mechanically removing the clot, but these methods are very invasive and carry their own risks. Devices have been developed to mechanically disrupt the clot, such as the penumbra system, which operates by aspirating and extracting the thrombus [3], and the Merci Clot Retrieval Device, which also mechanically removes the embolism and restores blood flow [4]. The Solitaire Revascularization Device is a stent-based device that allows immediate restoration of blood flow with the placement of a stent, followed by clot removal [5]. These surgical interventional techniques have shown benefit, but there are no double-blinded, randomized, controlled studies showing this benefit, and any studies conducted have only involved small patient numbers. While the methods for restoring blood flow to areas of ischemia are improving, we have yet to improve stroke outcome over what was first demonstrated in the NINDS trial in 1995 with IV tPA at <3 h after stroke onset [6].

The most critical factor affecting stroke therapy is that currently only 5% of patients are treated for stroke [1]. There are many factors which contribute to this fact, including a lack of public awareness of stroke symptoms, and lack of ability to reach appropriate care in time for current treatments to be effective. Due to these facts, there is a great need to develop additional therapies for patients at later time points, and for those who have a completed infarct. Stem cell based therapies are proving to be very promising candidates for treatments that are not only effective at later time points following stroke onset, but addressing the complex pathophysiology of stroke and providing neurological repair. This review will outline current preclinical and clinical research in the emerging field of stem cell therapeutics in stroke research. 

## 3. Stem Cell Therapies

Historically, the dogma put forth by Ramon Y Cajal that the total number of cells in the brain were fixed and that there was no cellular regeneration or presence of endogenous stem cells in the brain was widely accepted [7]. This dogma was accepted well into the 20th century, until studies by Altman and Das [8] provided evidence for the existence of stem cells in the brain. These early studies on neural stem cells as the basis for neurogenesis were subsequently confirmed by the Nottebohm group [9]. The discovery of neurogenesis in the brain lead to the discovery of neurogenic niches in the subventricular zone (SVZ) and the dentate gyrus (DG) [10]. Research in the field also revealed enhanced neurogenesis after injury in both neurogenic niches as well as non-neurogenic regions such as the cortex and striatum [11]. These discoveries have opened the door for investigating the potential use of endogenous neurogenesis as a treatment of stroke. 

### 3.1. Neuroregeneration

One of the potential mechanisms by which stem cells can provide therapy for stroke victims is through neuroregeneration. In order for stem cells to produce successful neuroregeneration, several conditions must be met: they must be able to produce multiple types of neurons and glia; the new cells must be able to migrate to the site of injury; and they must be able to integrate with existing circuitry by initializing and maintaining appropriate functional connections with neighboring cells. The adult brain is populated by endogenous stem cells known as neural stem cells (NSCs) which are capable of differentiating into different types of neurons and glia [12].

Many *in vivo* studies have demonstrated the production of both immature and mature neurons that migrate towards the striatum following a stroke. Ischemia-induced neurogenesis was first documented in the hippocampus 1998 [13]. This study showed an amplification of endogenous neurogenesis following global ischemia, but did not show any replacement of the CA1 pyramidal cells that are lost in ischemia. Similar amplifications of neurogenesis in neurogenic regions, such as the dentate gyrus and sub-ventricular zone, have been shown following focal ischemia [14,15]. One of the drawbacks of endogenous neurogenesis as a therapy for stroke is that the new cells have limited capabilities to migrate to the site of injury. Granulocyte colony stimulating factor (G-CSF) has arisen as a potential therapy to allow for the migration of endogenous stem cells to the site of ischemic injury [16].

Despite studies showing the ability to recruit endogenous new neurons to the site of injury, there are very few studies that have been able to show new neurons extending axons to appropriate targets, and there has been no evidence of existing neurons extending axons to new neurons *in vivo*. Given these results, we are still a long way from being able to use endogenous stem cells as a viable treatment for stroke injury.

### 3.2. Neuroprotection

Developments in the field of stem cell research have identified an application of exogenous stem cells as a vehicle for neuroprotective effects. One of the major benefits to these studies is that this approach to stroke therapy has shown to provide benefits far past the 3 h time window of current therapeutic approaches for stroke. 

Several labs [17,18,19], have tested various exogenous stromal stem cells intravenously in rodents after an induced stroke. These studies have revealed the potential for these types of treatment to provide multiple benefits, including reduced stroke volumes, improved functional outcomes, and an extended time window for treatment—up to 48 h following stroke onset. These studies in rodent models of stroke show great promise for future stroke therapies, due to their ability to improve stroke outcome and recovery, and most importantly, do so at a time point later than any currently available treatments.

Recent studies have focused on more specific cell types or stem cell populations that have been administered. Various mechanisms of delivery including intravenous (IV), intra-arterial (IA), and direct injection into the brain have been attempted. IV delivery of exogenous stem cells is desirable because it is the least invasive of all administration techniques. One drawback to this method is that many of the injected cells end up being caught in peripheral organs such as the liver, spleen and lungs. In phase I and II clinical trials, IV administration of autologous mesenchymal stromal cells were shown to be safe in both the short and long term [20,21]. IA delivery is more invasive, infusing cells directly into the artery that is perfusing the ischemic tissue. The benefit of this method is that it allows the cells to bypass any peripheral organs and go directly to the site of injury. Some concerns with this method of delivery are the potential for the injected cells to form microvascular occlusions, thus worsening the ischemia and resulting in higher mortality rates [22,23]. Finally, direct intra-cerebral injection is highly invasive and carries many risks, such as initial human studies showing adverse side effects such as seizures, subdural hematoma and worsening of motor function [24,25]. 

Despite the wealth of research in the field of therapeutic applications of stem cells, there is no unified theory for the mechanism of action exhibited by these cells. One of the longest held views on the mechanism of action of exogenous stem cells as a treatment for stroke is that administered cells migrate to the site of injury and are able to replace diseased and dead endogenous cells by engraftment and differentiation into functional neural and glial cells. Support for this theory comes from a number of studies in animal models, such as one by Hayase *et al*. [26], in which stem cells were injected into the cortex three days after stroke onset, and were found in the brain 100 days later, with evidence of projections and expressing positive markers for glutamatergic, dopaminergic, and GABAergic neurons. The animals that were injected with these cells also showed significant functional recovery. 

The evidence for engraftment and transdifferentiation, however, has been heavily scrutinized, and many studies have provided evidence against this theory of stem cells as the sole mechanism of action. It has been shown that adult hematopoietic stem cells cannot differentiate into functional neurons. One of the pitfalls many studies face is that they look for neuronal markers for evidence of transdifferentiation, but do not test the engrafted cells for functionality. A study by [27] showed that when hematopoietic stem cells were grown in culture, they were able to differentiate into neuron-like cells, but these cells were not capable of producing an action potential. When these same cells were transplanted into the brain, there was no evidence of a neuronal phenotype. Instead, the injected cells differentiated into microglia, or died shortly after transplantation. This study, and many others like it [19], have shown that while there are often significant improvements in stroke outcome following stem cell administration, and engraftment and transdifferentiation may not be the mechanism by which these cells are acting.

An emerging theory as to the mechanism of action of therapeutic stem cells has arisen from a large number of studies that have shown a beneficial effect of the administration of stem cells following stroke, but have found little evidence of extensive engraftment or cell replacement in the brain. It has been suggested that the mechanism by which exogenous stem cells act is by altering the local immune response at the site of injury and modulating chronic inflammation. The tissue surrounding any type of brain injury has been shown to be highly pro-inflammatory, with increases in pro-inflammatory cytokines such as IL-1a, IL-1b, IL-6, and TNF-α upregulated at 48 h following injury [28]. 

Several studies have shown that administration of exogenous stem cells into a stroke animal have resulted in a reduction in inflammatory cytokines, as well as an upregulation in anti-inflammatory cytokines. Liu *et al.* [29] showed that MSC that were injected into the cortex following stroke in a rat model not only decreased the infarct size, but that IL-10 was up regulated and TNF-α was down regulated following MSC administration, suggesting an anti-inflammatory effect of the MSCs. 

An *in vitro* study of MSCs grown in contact culture with NSCs showed an increase in IL-6 production as well as a decrease in apoptosis. These results suggest that the direct implantation of MSCs that come into contact with endogenous NSCs stimulates the local immune response through NFkB activity [30]. This result was not replicated in *in vitro* studies without cell-cell contact.

When looking to apply cell therapies in the clinic, opting for less invasive therapies is preferable. IV and IA administration of stem cells have been studied in many animal models of stroke and brain injury. These studies generally show little to no cell engraftment in the brain, but do show decreases in infarct volume as well as improvements in functional outcome measures. One common observation is that this type of administration results in what is known as the “pulmonary first pass effect” [31]. IV administration results in the majority of injected cells becoming caught in the lungs, spleen, kidney, and liver. Yet significant infarct reduction and improvement in functional recovery has been repeated in numerous studies. 

One suggested mechanism of action in these instances is modulation of the systemic immune response which stimulates anti-inflammatory and pro-survival responses that ameliorate stroke injury. There is evidence that systemically administered stem cells interact with immune cells in multiple organ systems. For example, stem cells that become caught in the lungs have been shown to interact with pulmonary macrophages and modulate the systemic inflammatory response [32]. As previously discussed, modulation of the inflammatory response is key in improving stroke outcome. It has also been shown that IV administration of MSCs results in a decrease in the pro-inflammatory cytokines TNF-α and IL-6 in the serum, as well as an increase in the anti-inflammatory cytokine IL-10 [32]. Systemically administered stem cells can also interact with splenocytes to have an effect on the overall immune response following stroke. A study by [33], systemically administered NSCs in ischemic rats, resulting in improved functional outcomes and reduced infarct size, though very few transplanted cells were found in the cortical tissue. Cytokine analysis showed a decrease in the pro-inflammatory cytokines TNF-α and IL-6 in both the brain and the spleen, and histology showed a large number of NSCs present in the splenic tissue. Stroke animals receiving NSCs that had splenectomies did not show any improvement following ischemic injury, providing a strong case for the necessity of NSC interaction with splenocytes for improved stroke recovery.

Alterations in the pro- and anti-inflammatory cytokine profiles of stroke animals as a result of stem cell therapy may be crucial to ameliorating stroke deficits. In addition to affecting the inflammatory profile, stem cells can secrete cytokines that promote angiogenesis and neovascularization [34]. It is, perhaps, by altering the local and systemic immune system that provides the benefit that is seen following stem cell administration, even when no engraftment occurs. 

## 4. Stem Cell Transplant for Treatment of Stroke

### 4.1. Goals for Stem Cell Transplant

In order for cell transplantation to successfully provide therapy, cells must either cross the blood brain barrier and influence the local stroke milieu, influence the systemic immune response, or replace cells lost to ischemia, resulting in improved outcome and reduced injury. If used to generate new neurons, these new neurons must mature, form synaptic connections and not die. If used for neuroprotection, then it will be important to understand if it is needed for them to cross the blood brain barrier, as well as understanding the effect of first pass through the lungs and the liver. It will be important to know the drawbacks and advantages of each type of cell administration: IV, IA, and direct injection. It must also be determined whether or not it is better to use autologous cells which likely will require time before given to a stroke patient or to have banked cells that can be given immediately. 

### 4.2. Stem Cell Types

#### 4.2.1. Embryonic Stem Cells (ESCs)

Embryonic stem cells (ESCs) have long been considered the gold standard for pluripotency, and are considered ideal for therapy due to their ability to differentiate into any cell type including neurons and glia. One of the major drawbacks of using this cell type is that they can create teratomas if they are injected in an undifferentiated state [35]. The post-ischemic environment has been indicated as a cause of teratoma formation from undifferentiated cells, yet hyperproliferation has also been documented in cells that were injected at a later stage of differentiation, and this effect is independent of the surrounding environment [35]. It is very difficult to create a homogenous culture of ESCs that are completely without undifferentiated cells that may cause teratoma formation. It is critical that any graft used for therapy in humans be devoid of undifferentiated cells. These cells also are surrounded by political and ethical concerns that have limited research and would make them more difficult to apply to the clinical setting. 

Several studies have shown ESCs transplanted into the ischemic brain that exhibit neuronal markers and synaptic connectivity. [36] injected ESC derived neural precursors into mouse cortex and found the transplanted cells survived up to 12 weeks, although the number of surviving cells was much lower than the number that were injected. The surviving transplanted cells differentiated into glial cells and neurons of several different neurotransmitter producing types. Similar evidence of the functionality of transplanted ESCs has been shown by [37], in which NPCs derived from monkey ESCs were injected into MPTP monkeys—a primate model of Parkinson’s Disease. They found that the transplanted cells were able to function as dopaminergic neurons. In addition, animals receiving the cell transplant exhibited improved behavioral recovery. A recent study showed that transplantation of ES-NPCs into healthy murine cerebral cortex results in manifestation of dendritic and axonal connections. In addition, these transplanted cells did not form inappropriate connections [38]. 

#### 4.2.2. Inducible Pluripotent Stem Cells (iPSCs)

The reprogramming of adult fibroblast cells to exhibit properties of embryonic stem cells, has led to the generation of so-called inducible pluripotent stem cells (iPS cells). Until recently, issues surrounding immune rejection, potential for the formation of tumors, and ethical concerns decreased the attraction of pursuing stem cell transplantation as a therapy for stroke. However, landmark studies by Yamanaka identifying iPS, or inducible pluripotent stem cells, provided a promising new direction for stem cell therapy. iPS cells are derived by genetically reprogramming adult fibroblasts into an embryonic stem cell-like state by expressing a set of genetic factors that allow the cells to exhibit pluripotency [39,40]. These cells are being developed in several therapeutic settings due to their ability to differentiate into cells that are characteristic of all three embryonic germ layers. An additional benefit of these reprogrammed cells as cell therapies is that fibroblasts from patients can be taken and reprogrammed, thus eliminating the need for immune suppression. 

Since the initial study by Yamanaka and his colleagues, many studies have emerged on the production of iPS cells using various combinations of factors and generating iPS cells from various tissue sources. Due to the limited availability of these cells, very few stroke-specific studies have been conducted using iPS cells as therapy. Kawai *et al*. [40] studied the administration of iPS cells in a rodent model of stroke, and found severe teratoma formation in the brains of both the stroke and sham operated animals. 

These results show similar caveats to the use of ESCs for therapy—*i.e.*, the administration of undifferentiated iPS cells may result in teratoma formation, making them unsuitable for therapy in their undifferentiated state. A recent study has demonstrated that the epigenetic changes induced in the process of generating iPS cells can induce immune rejection [41]. Whether this will occur in clinical practice, however, remains unknown. While iPS cells can be expanded and can be used to produce various cell types, it remains difficult to generate highly purified cell populations and even when directed to differentiate into specific neuronal populations there is still a significant potential to form teratomas *in vivo* [42]. 

#### 4.2.3. Neural Stem Cells (NSCs)

Another type of cell that has been investigated for its therapeutic properties is the neural stem cell (NSC). These cells can be isolated from several regions (the sub-ventricular zone and the sub-granular zone) in the central nervous system of embryos as well as in adults and can be grown and expanded in culture as neurospheres [14]. Unlike ESCs, NSCs are not pluripotent, but they can differentiate into many, but not all, types of CNS cells. They have been shown to differentiate into neurons, astrocytes, and oligodendrocytes. While some studies have used grafts of exogenous NSCs, others have attempted to recruit endogenous NSCs to the site of injury as a potential therapy. A study by Li *et al*. [43], demonstrated that the endogenous NSCs in the adult brain that are activated by ischemia are necessary for neuroprotection of the ischemic brain. By inhibiting cell proliferation in the brain following ischemia, they found a large increase in infarct size and functional deficit, indicating a crucial role for endogenous NSCs in stroke recovery. Another study has shown that the intra-ventricular administration of NSCs results in improved functional recovery at 28 days following stroke, and also found that NSCs expressing HIF-1a further improved recovery [44]. While many studies have shown the potential therapeutic benefit of both endogenous and transplanted NSCs, one of the limiting factors of this type of treatment is the poor survival rate of transplanted cells. It has been reported that as little as 1%–3% of the transplanted cells survive, and an even smaller percentage have been shown to differentiate into neurons [45]. Despite the low survival rate, studies of NSCs have shown consistent therapeutic benefits in animal models of ischemic stroke. 

### 4.3. Adult Tissue-Derived Stem Cells

One of the most popular sources of cells for cell-based therapy is adult tissue-derived stem cells. These include, but are not limited to, bone marrow derived stem cells, umbilical cord blood derived stem cells, and teratocarcinoma cells. Bone marrow derived cells are among the most commonly researched adult stem cell types, and were identified as potential therapeutic targets due to the endogenous behavior of bone marrow cells. 

Marrow Stromal Cells (MSCs) are a type of bone marrow derived stem cell that have been widely studied for their therapeutic benefits. It has been demonstrated that these cells migrate towards tissue injury signals *in vitro* [46], however, *in vivo* models have shown a very poor survival rate of transplanted MSCs [47]. Despite the low rate of cell survival following transplantation, many studies have shown improved outcome following ischemic injury after the transplantation of MSCs [48]. A second type of bone marrow derived stem cell is multipotent adult progenitor cells (MAPC). These cells were originally thought to be superior to MSCs due to their ability to generate functional cell types from all three embryonic germ layers, including neurons with mature electrophysiological properties [49]. However, studies of transplantation of MAPCs resulted in improved recovery from ischemia [50], but did not show robust evidence of engraftment [51]. 

Another type of adult-derived stem cells are teratocarcinoma cells, which are a variant of embryonic stem cells that have been derived from an immortalized cell line of germ cell tumors. These cells are able to differentiate into a pure population of neurons when exposed to retinoic acid [52]. These cells have been tested in clinical trials [24] as a treatment for stroke and shown to be safe as well as exhibit engraftment as postmitotic neuronal cells. The patients in this study showed a small amount of functional improvement, but this improvement was not found to be statistically significant [18]. Although these cells avoid any ethical concerns such as those that arise from ESC use, questions of safety will always be a concern with this cell line, due to the tumorigenic nature of the donor cells. 

A promising source of therapeutic adult-derived stem cells is umbilical cord blood, which has been shown to be rich in stem cells, varying fractions of which have been tested in animal models of ischemic stroke as a potential therapy. Administration of these cells has shown a dramatic decrease in infarct size as well as improved functional recovery [17,18,19]. A recent study combining the administration of Simvastatin in addition to human umbilical cord blood stem cells in a rat model of stroke showed an increase in injected cells migrating into the brain. As a result, they showed increased neural plasticity and improved neurological outcome [17]. Many studies using umbilical stem cells show little to no evidence of surviving transplanted cells in the brain following systemic delivery, suggesting that cell replacement is not the mechanism of action for the beneficial effects of these cells. 

An extension of the concept of reprogramming somatic cells to become iPSCs is the method of direct reprogramming to generate neurons directly from adult fibroblasts. Recent studies have demonstrated the production of several neuronal types through the approach of direct reprogramming [53]. Since these cells do not re-enter the cell cycle, there is no potential to form tumors. One of the limitations of using these types of cells as a therapy for stroke is that it is difficult to generate a sufficient number of cells; generation of directly reprogrammed cells takes time, and therefore may not be produced in time for administration when needed.

As described previously, many types of adult derived stem cells exhibit an innate tropism towards a site of injury. Some researchers are harnessing the ability of these cells to migrate towards injury sites and reprogramming them to act as vehicles of gene therapy. Adult stem cells can be made to deliver therapeutic molecules that are anti-inflammatory, pro-angiogenic, pro-survival, or anti-apoptotic [54]. MSCs have been modified to express PlGF (Placental Growth Factor), which is angiogenic to impaired non-neural tissue. Stroke animals that received MSCs expressing PlGF showed a decrease in infarct size, as well as an increase in functional recovery and angiogenesis at the site of injury [55]. Another study utilized MSCs expressing Ang-1, and found that the stroke animals that received the genetically modified cells showed an increase in angiogenesis and neovascularization, specifically at the border of the infarct area, as well as overall increased cerebral blood flow and a decrease in infarct size [56]. These studies suggest that gene therapy using stem cells as vehicles may provide added benefit and improved outcome following ischemic stroke. 

### 4.4. Stem Cell Conditioned Media

Given the strength of the evidence supporting the theory that the benefits of stem cell therapy for stroke comes from expressed factors, such as cytokines, chemokines, and growth factors, has led a number of research groups to investigate whether the same benefits can be observed with the administration of stem cell conditioned media. Conditioned media is desirable as a potential therapy because it would avoid some of the limitations posed by cell-based therapy, such as the low survival rate of implanted cells, potential for teratoma formation, and immune rejection of injected cells. 

Conditioned medium from human adipose stem cells has been shown to provide therapeutic effects in an animal model of stroke. This study showed that a continuous administration of the cultured media for seven days following stroke resulted in a reduction in infarct volume, improved functional recovery, and a significant reduction in microglial apoptosis and astrogliosis [57]. Stem cell-cultured media has also been shown to provide therapeutic benefits in disease models other than stroke. Human umbilical cord blood-derived mesenchymal stem cells have been shown to protect neurons against amyloid-beta neurotoxicity. Secretion of decorin and proganulin [58], or soluble intracellular cell adhesion molecule 1 [59] by cord blood mesenchymal stem cells have been shown to protect against neuronal death in tissue culture and *in vivo*. Human embryonic mesenchymal stem cell-derived conditioned medium has also been shown to rescue kidney function and reduce hypertension in a rat model of chronic kidney disease. The evidence from this study suggests that the protective effects of the conditioned media are due to increased endothelial cell regeneration due to active DNA damage repair, as well as angiogenesis [60]. Additionally, MSC-conditioned media has been shown to improve recovery from acute lung injury in mice. This study showed that the administration of the MSC-conditioned media promotes alternative macrophage activation to a wound healing and anti-inflammatory M2 phenotype [60,61]. 

These studies show that stem cell-conditioned media does have a therapeutic effect in multiple disease models. However, there is much that still needs to be determined before conditioned media can be considered as a viable therapeutic agent. Further studies must be conducted to fully profile the secreted factors produced by various types of stem cells. In addition, the ways in which this profile changes due to various types of stimulation (*i.e.*, cell to cell contact) must be fully characterized. The data from these studies clearly shows that secreted cytokines, chemokines, and growth factors play a large role in the therapeutic mechanism of action of exogenous stem cell administration. 

## 5. Clinical Trials

The promising results of preclinical research in this field have prompted a large number of preliminary clinical studies to be launched in recent years. One of the earlier clinical trials to investigate the efficacy of stem cells as a treatment for stroke tested the safety and efficacy of mesenchymal stem cells (MSCs) in a randomized and controlled phase I/II trial [21]. This study recruited patients that had acute cerebral infarction within the area of the middle cerebral artery, and aspirated bone marrow from these patients one week following stroke onset. MSCs were isolated from the marrow and administered intravenously between five and seven weeks following stroke. Short term follow up showed no adverse effects from the cell administration, and functional recovery improved in patients receiving MSC therapy according to the Barthel Index and the modified Rankin Score. A long term follow up study of the same patients confirmed that intravenous delivery of MSCs is safe, and that some patients may derive a functional benefit, though they noted that functional improvement was associated with serum levels of stromal cell-derived factor-1, as well as the involvement of the subventricular region of the lateral ventricle [20].

A second clinical trial also tested the effects of bone marrow-derived mononuclear cells as a treatment for stroke [62]. In this study, patients were administered intra-arterially between 5 and 9 days following stroke. This study found no adverse effects related to the cell transplantation. However, there was also no significant neurological improvement at 180 days following the cell administration. There was a trend towards functional improvement in the Barthel Index correlating with the number of CD34+ cells that were injected. 

Of the many ongoing clinical trials in this field, the majority focus on determining the safety and efficacy of bone marrow derived stem cells as a treatment for stroke. Other cell types being studied include: modified stem cells, umbilical cord blood derived stem cells, and adipose derived stem cells. The majority of the ongoing clinical trials are administering the therapeutic cells systemically either via IV or IA administration, as this method of delivery is the least likely to cause complications. The studies encompass therapies for both chronic and acute ischemic stroke. A summary of current ongoing stem cell trials for stroke can be found at the website ClinicalTrials.gov and are listed for convenience below in Table 1. At the present time, the results of these current studies have yet to be reported and are anxiously awaited.

**Table 1:** Ongoing clinical trials of stem cell therapies for ischemic stroke.

Stem cell type	Administration	Therapeutic time point following stroke	Clinical trial ID
Autologous PBSC (CD34+)	Intercerebral Implantation	Na	NCT00950521
Mesenchymal stem cells	IV	<6 weeks	NCT00875654
Autologous bone marrow stem cell	IV	7–30 days	NCT01501773
Autologous bone marrow stem cell (CD34+)	Infusion into MCA	5–9 days	NCT00761982
Autologous BMSC	IV	5 weeks	NCT01468064
Autologous bone marrow CD34+	Infusion into MCA	<7 days	NCT00535197
Autologous bone marrow mononuclear stem cell	IV	24–72 h	NCT00859014
CTX0E03 neural stem cells	Local injection	6 months–5 years	NCT01151124
SB623 modified stem cell	Na	6–36 months	NCT01287936
Allogenic adult mesenchymal bone marrow stem cells	IV	>6 months	NCT01297413
MultiStem	IV	1–2 days	NCT01436487
Allogenic CD34+ umbilical cord blood stem cells	Brain Implant	6–60 Months	NCT01438593
Autologous peripheral hematopoietic stem cell	Cerebral artery transplant	<1 year	NCT01518231
Autologous human umbilical cord blood	IV	6 weeks–6 years old, history of prenatal stroke	NCT01700166
Allogenic mesenchymal stem cells from adipose tissue	IV	<12 h	NCT01678534
Adipose-derived stem cells	IV and ICA	Na	NCT01453829
Autologous bone marrow stem cells	IA/IV	>3 days and <90 days	NCT00473057
Autologous BMMCs and marrow stromal cells	IV	<72 h	NCT00908856
*Ex-vivo* cultures adult allogenic MSCs	IV	<10 days	NCT01091701

## 6. Conclusions

There is a wealth of evidence supporting the use of stem cell therapies for ischemic stroke, but the mechanisms by which these cells exert their neuroprotective effects have yet to be fully elucidated. It must also be determined if different cell types have distinct mechanisms of action, as well as what risks are posed by each cell type and delivery method. With a more complete understanding of the mechanisms by which cell based therapies are able to ameliorate stroke injury, we will be able to successfully transition these therapies from the lab bench into the clinic. 
